# Supplementary material for: L-Serine Supplementation Blunts Fasting-Induced Weight Regain by Increasing Brown Fat Thermogenesis
Source: Nutrients. 2022 May 4;14(9):1922. doi: 10.3390/nu14091922 (PMC9104834; doi:10.3390/nu14091922)
Supplement: Supplementary file 1 [file nutrients-14-01922-s001.zip › Supplementary Information Lopez Gonzales.pdf]

**Supplementary Information**

**Table S1: qPCR primers**

| | Forward | Reverse |
|---|---|---|
| **CD68** | TCACCTTGACCTGCTCTCTC | AGGCCAATGATGAGAGGCAG |
| **IL-6** | GTCGGAGGCTTAATTACACATGT | CAAGTGCATCATCGTTGTTCA |
| **Npy** | ACCGGTGGTCTCTTCTCTCA | CTTGTTACCTAGCATCGTGGC |
| **Pepck** | CTGCATAACGGTCTGGACTTC | CAGCAACTGCCCGTACTCC |
| **Pgc1α** | AGCCGTGACCACTGACAACGAG | GCTGCATGGTTCTGAGTGCTAAG |
| **Pomc** | GCGAGGCAAACAAGATTGG | GAGGCACTGAACATCTTTGTC |
| **Prdm16** | CCGCTGTGATGAGTGTGATG | GGACGATCATGTGTTGCTCC |
| **Tbp** | ACCCTTCACCAATGACTCCTATG | TGACTGCAGCAAATCGCTTGG |
| **TNFα** | CCCACGTCGTAGCAAACCA | GTCTTTGAGATCCATGCCGTTG |
| **Ucp1** | CTGCCAGGACAGTACCCAAG | TCAGCTGTTCAAAGCACACA |

**Supplementary Figure 1. L-serine supplementation does not affect white adipose (WAT) tissue inflammation or cell size in ad libitum fed mice.**

**(a)** Body weight of ad libitum CD and HFD fed mice with or without L-serine supplementation starting at 8 weeks of age (CD: *n*=5; HFD: *n*=4; CD+L-ser, HFD+L-ser: *n* =6). **(b)** Expression of inflammatory marker genes in SCF and PGF of CD and HFD fed mice with or without L-serine supplementation after 8 weeks starting at 4 weeks of age (*n*=8). **(c)** Adipocyte size distribution of subcutaneous fat (SCF) and perigonadal fat (PGF) of random fed mice in CD and HFD with or without L-serine supplementation for 8 weeks starting at 4 weeks age (*n*=3 animals >10000 cells per tissue and mouse). Adipocytes were grouped in different size categories. (**d**) Triglyceride content of liver from CD and HFD fed mice with or without L-serine supplementation for 8 weeks starting at 4 weeks of age **(e)** Serine content in liver of CD or HFD fed mice with or without L-serine supplementation for 8 weeks starting at 4 weeks of age (*n*=8). **(f)** Liver weight of CD or

HFD fed mice with or without L-serine supplementation for 8 weeks starting at 4 weeks of age (*n*=8). **(g)** Glycogen levels in livers of CD or HFD fed mice with or without L-serine supplementation for 8 weeks starting at 4 weeks of age (*n*=8). **(h)** Pepck gene expression in liver of CD or HFD fed mice with or without L-serine supplementation for 8 weeks starting at 4 weeks of age (*n*=8). **(i)** Pyruvate Tolerance test (PTT) of CD and HFD fed mice with or without L-serine supplementation after 1 week starting at 8 week of age (2mg/kg pyruvate; *n*=6). Data are shown as mean ± SEM, one or two way ANOVA with Tukey's post-hoc test. $^{**}p<0.01$.

**Supplementary Figure 2. L-serine supplementation reduces white adipocyte size and white adipose tissue (WAT) inflammation upon fasting.**

**(a)** Liver weight of CD and HFD fed mice with or without L-serine supplementation after 8 weeks and repeated overnight fasting starting at 8 weeks of age (n=6). **(b)** Triglyceride content 8 weeks (*n*=6). (**c**) Representative H&E stainings of liver after 8 weeks. **(d)** Adipocyte sizes of SCF and PGF after 8 weeks and repeated overnight fasting starting at 8 weeks of age (*n*=4 mice; >7000 adipocytes per group for PGF and >10000 adipocytes per group for SCF). Statistical analysis using a one-way ANOVA with a Tukey's post-hoc test revealed statistically significant differences between all groups in SCF and PGF. (**e**) Expression of inflammatory marker genes in PGF and SCF of fasted CD and HFD fed mice with or without L-serine supplementation after 8 weeks and repeated overnight fasting starting at 8 weeks of age (CD, HFD, HFD+L-ser : *n*=6; CD+L-ser: *n*=4-5). Data are shown as mean ± SEM, one way ANOVA with Tukey's post-hoc test. * $p<0.05$, ** $p<0.01$.

**Supplementary Figure 3. L-serine supplementation increases cumulative high fat diet intake in fasted mice.**

**(a)** Experimental setup for 16-week L-serine supplementation and indirect calorimetry measurement (TSE) in CD and HFD fed mice with and without L-serine supplementation. The figure was generated using Biorender.com. **(b)** Cumulative food intake of random fed and fasted CD and HFD fed mice with or without L-serine supplementation, starting at 8 weeks of age, at week 7(CD) or 8 (HFD) (CD random fed: *n*=5; CD fasted, CD+L-ser random fed, CD+L-ser fasted: *n*=6; HFD random fed: *n*=4; HFD fasted, HFD+L-ser random fed, HFD+L-ser fasted: *n*=6). **(c)** Average water intake (g) day total, light and dark phases of random fed and fasted CD and HFD fed mice with or without L-serine supplementation, starting at 8 weeks of age, at week 7(CD) or 8 (HFD) (CD random fed: *n*=5; CD fasted, CD+L-ser random fed, CD+L-ser fasted: *n*=6; HFD random fed: *n*=3; HFD fasted: *n*=5;HFD+L-ser random fed, HFD+L-ser fasted: *n*=6). **(d)** mRNA expression of NPY and POMC from the hypothalamus of random fed and fasted CD and HFD fed mice with or without L-serine supplementation starting at 8 weeks of age, at week 16 (CD random fed: *n*=4; CD fasted, CD+L-ser random fed, CD+L-ser fasted: *n*=6; HFD random fed: *n*=4; HFD fasted, HFD+L-ser random fed, HFD+L-ser fasted: *n*=6). **(e)** Respiratory exchange ratio (RER) of random fed and fasted mice at week 7(CD) or 8 (HFD) (CD random fed: *n*=5; CD fasted, CD+L-ser random fed, CD+L-ser fasted: *n*=6; HFD random fed: *n*=4; HFD fasted, HFD+L-ser random fed, HFD+L-ser fasted: *n*=6). **(f)** Western blots of phosphorylated-HSL 563, Ser660, Ser565 and total HSL in PGF and SCF of random fed and fasted CD or HFD fed mice with or without L-serine supplementation for 8 weeks starting at 8 weeks of age (*n*=6). Data are shown as mean ± SEM, two way ANOVA with

Tukey's post-test. Data are shown as mean ± SEM, two way ANOVA with Tukey's post-hoc test. *$p<0.05$, ** $p<0.01$, $p$***<0.001, $p$****<0.0001.

**Supplementary Figure 4. L-serine supplementation does not alter thermogenic gene expression in brown adipose tissue (BAT).**

**(a)** Average locomotor activity (counts) of random fed and fasted CD and HFD fed mice with or without L-serine supplementation at week 7(CD) or 8 (HFD) (CD random fed: $n$=5; CD fasted, CD+L-ser random fed, CD+L-ser fasted: $n$=6; HFD random fed: $n$=4; HFD fasted, HFD+L-ser random fed, HFD+L-ser fasted: $n$=6). **(b)** BAT weight of fasted CD and HFD fed mice with or without L-serine supplementation for 8 weeks starting at 8 weeks of age ($n$=6). **(c)** mRNA expression of thermogenesis-related genes in BAT after 8 weeks starting at 8 weeks of age ($n$=6). **(d)** Western blots for UCP1 of random fed and fasted CD or HFD fed mice with or without L-serine supplementation for 8 weeks starting at 8 weeks of age. Data are shown as mean ± SEM, one or two way ANOVA with Tukey's post-hoc test. *$p<0.05$.
