# Supplementary figures and images for: L-Serine Supplementation Blunts Fasting-Induced Weight Regain by Increasing Brown Fat Thermogenesis
Source: Nutrients. 2022 May 4;14(9):1922. doi: 10.3390/nu14091922 (PMC9104834; doi:10.3390/nu14091922)

Supplementary Figure 1

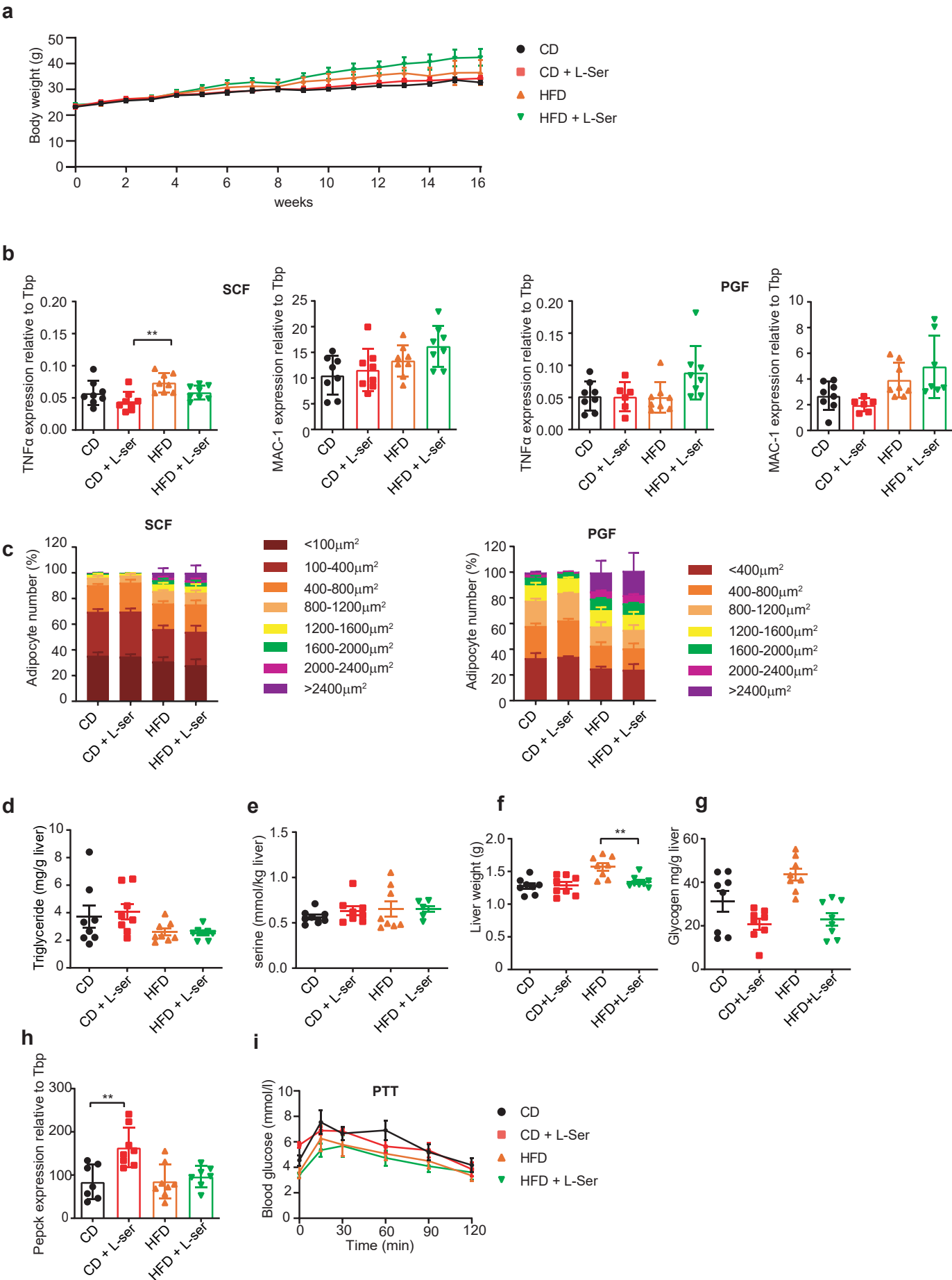

Supplementary Figure 2

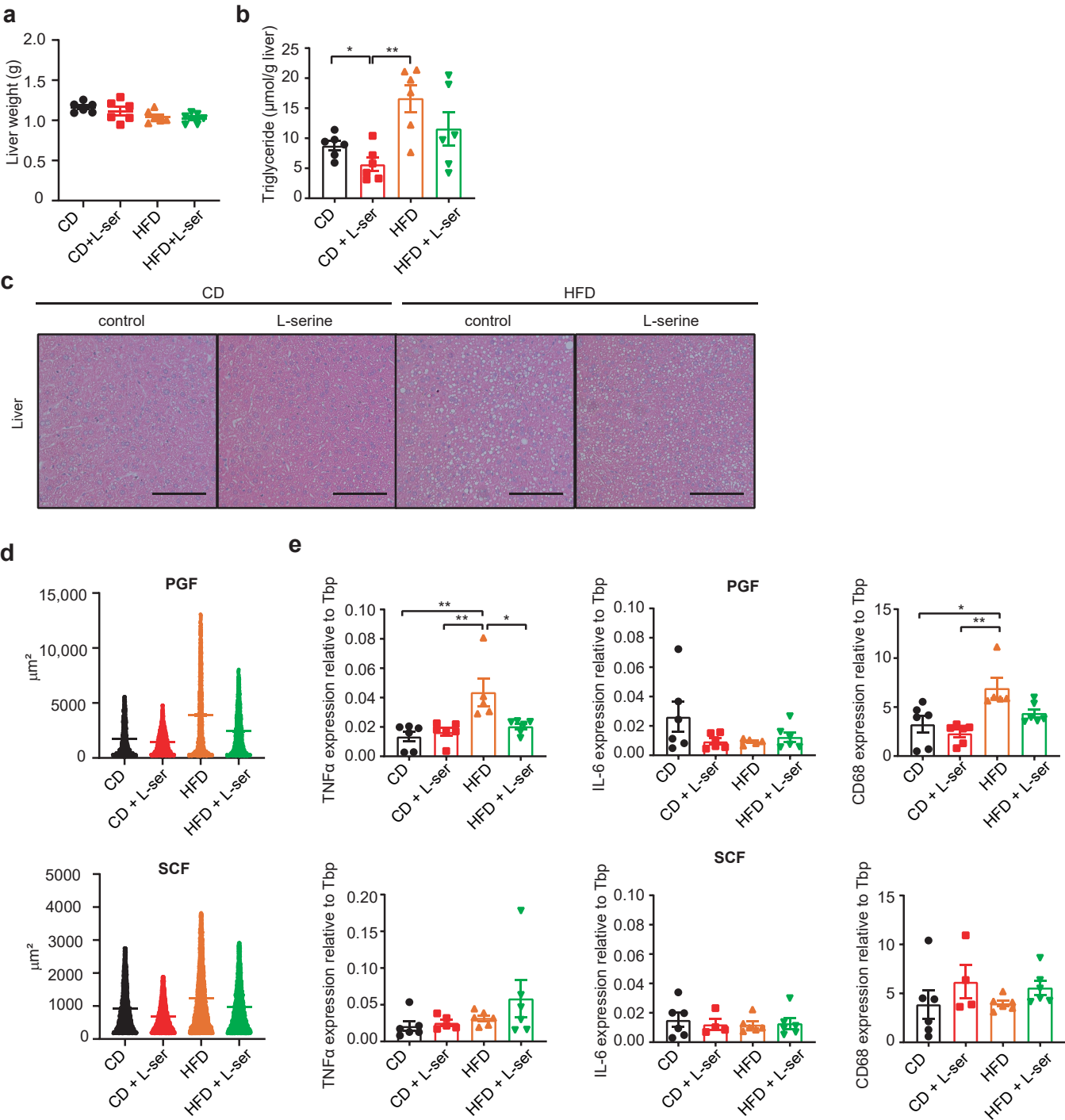

Supplementary Figure 3

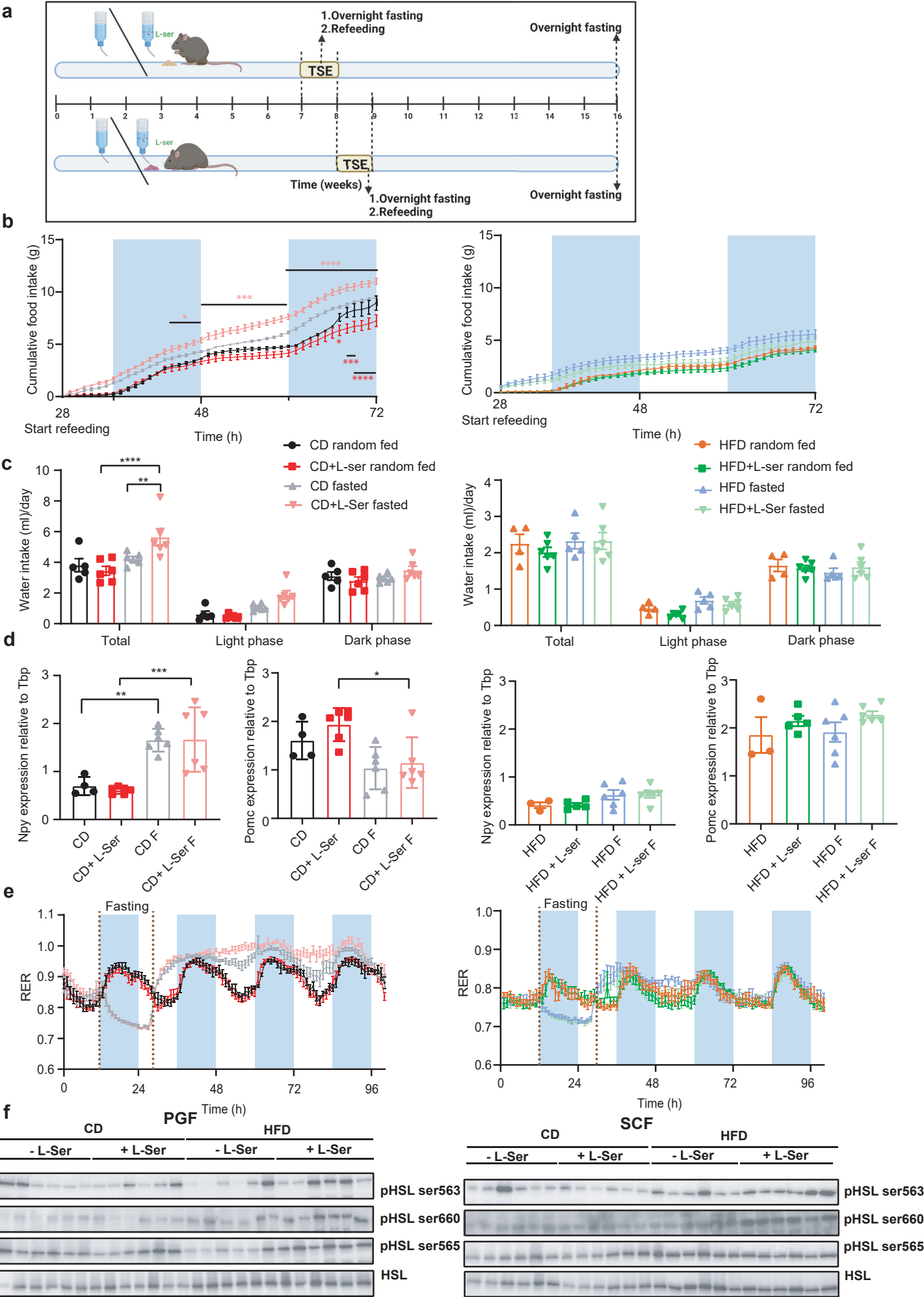

Supplementary Figure 4

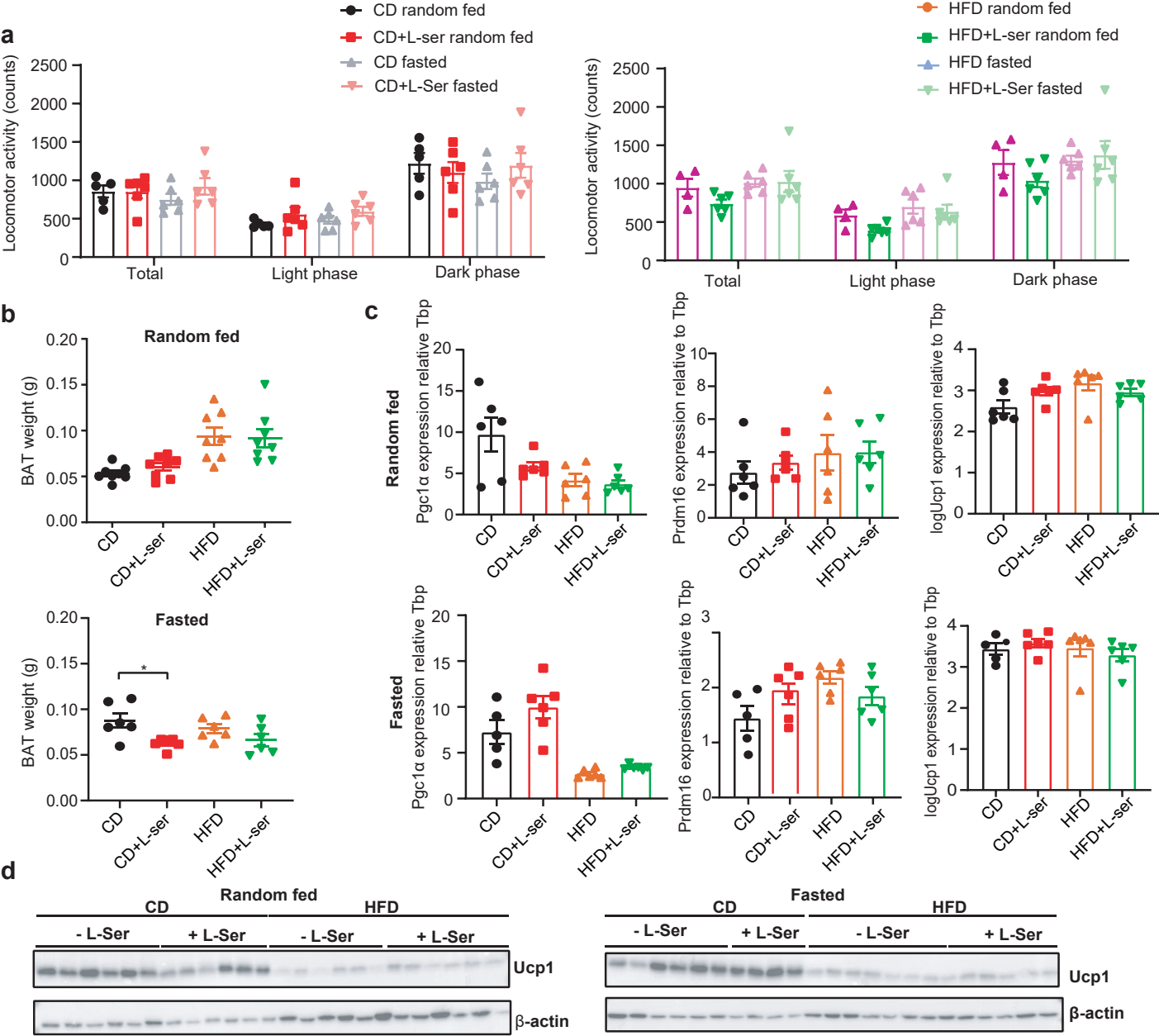

Supplement: Supplementary file 1 [file nutrients-14-01922-s001.zip › L-serine_Supplemental figures_Proof.pdf]
